# Herpes hepatitis as a complication of total abdominal hysterectomy; an unusual complication of abdominal instrumentation

**DOI:** 10.1002/ccr3.1890

**Published:** 2018-11-08

**Authors:** Muharrem Yunce, Pavan Bhat, Daisy Jaganathan, Michelle Bahrain

**Affiliations:** ^1^ Department of Medicine MedStar Franklin Square Medical Center Baltimore Maryland; ^2^ Department of Infectious Disease MedStar Franklin Square Medical Center Baltimore Maryland

**Keywords:** herpes hepatitis, immunocompetent, post‐operative complication

## Abstract

Herpes simplex virus hepatitis is a rare but potentially fatal disease without early intervention. Impaired immunity is a major predisposing risk factor but infection in immunocompetent individuals is not unheard of. Diagnosis is complicated by its rarity and nonspecific signs and symptoms on presentation. Identification by liver biopsy is often limited due to concurrent coagulopathy. Early and aggressive treatment is centered on antiviral therapy with acyclovir. We present a case of herpes hepatitis in an immunocompetent woman following abdominal instrumentation.

## 1 CASE PRESENTATION

A 36‐year‐old woman was admitted to the hospital for laparoscopic robotic hysterectomy and bilateral salpingectomy for high‐grade squamous intraepithelial lesion found on screening pap smear and proven to be cervical intraepithelial neoplasia 2‐3 on colposcopy. The patient was stable throughout surgery and was discharged home with no complications.

**Figure 1:**
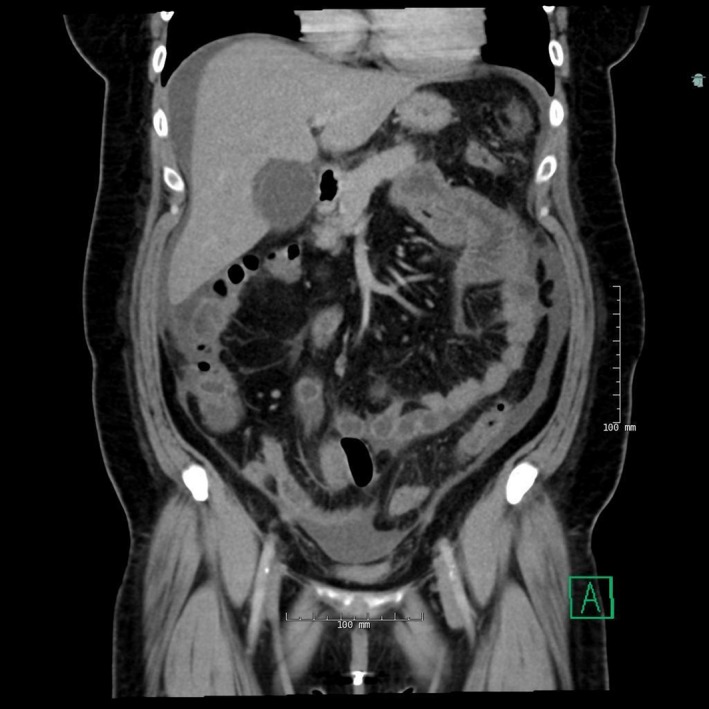
CT abdomen and pelvis coronal view demonstrating minimal ascites around liver

**Figure 2:**
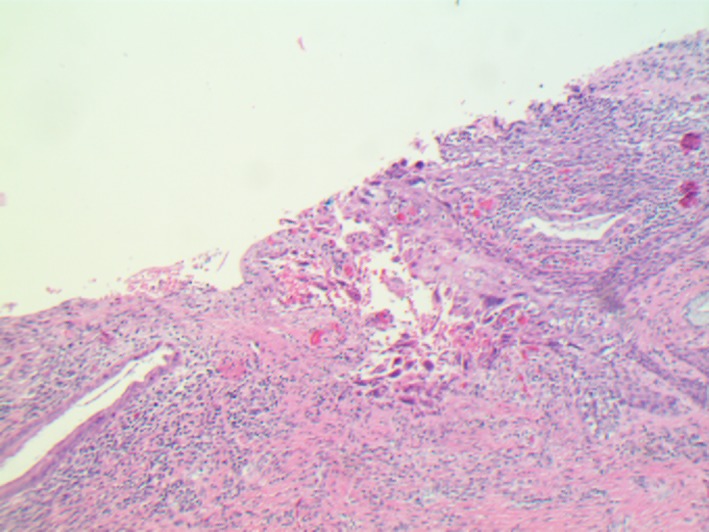
Herpes cervicitis gross appearance (10x)

**Figure 3:**
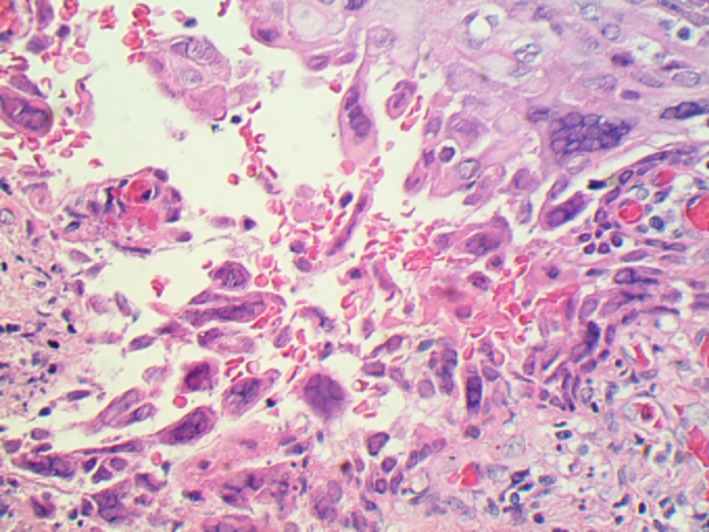
Herpes cervicitis (40x) showing classic HSV viral cytopathic effects (multinucleated cells with chromatin margination)

The patient presented to emergency department 3 days after surgery with abdominal pain, nausea, vomiting, and fever of 101°F. Upon presentation to ED, temperature was 37.5°C, heart rate 93 per minute, blood pressure 124/62, SpO_2_ of 95% on room air, respiratory rate of 20. Physical exam was consistent with mild tenderness on palpation throughout the lower abdomen without peritoneal signs.

Laboratory data revealed WBC of 3.8 (lowest 1.6), platelet of 86 (lowest 66), AST of 2310 (max), and ALT of 686 (max).

Computerized tomography of abdomen and pelvis revealed small amount of ascites around the liver and spleen extending into the pericolic gutters bilaterally with fat stranding within the omentum (Figure 1).

The patient was started on piperacillin/tazobactam with a presumptive diagnosis of postoperative peritonitis. Interestingly, the patient's pathology from the cervical tissue was positive for herpes simplex virus (HSV) (Figures 2 and 3). The patient continued to be febrile which prompted a paracentesis. This fluid was sent for viral PCR and demonstrated positive result for HSV. The test was not able to differentiate between HSV type 1 vs 2.

**Graph 1:**
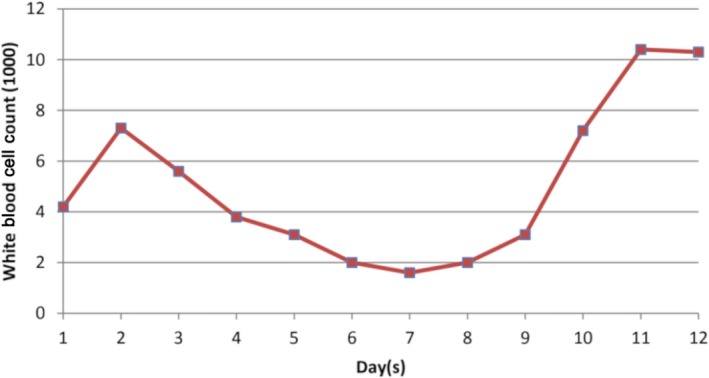
Trend of white blood cell count over the course of hospitalization

**Graph 2:**
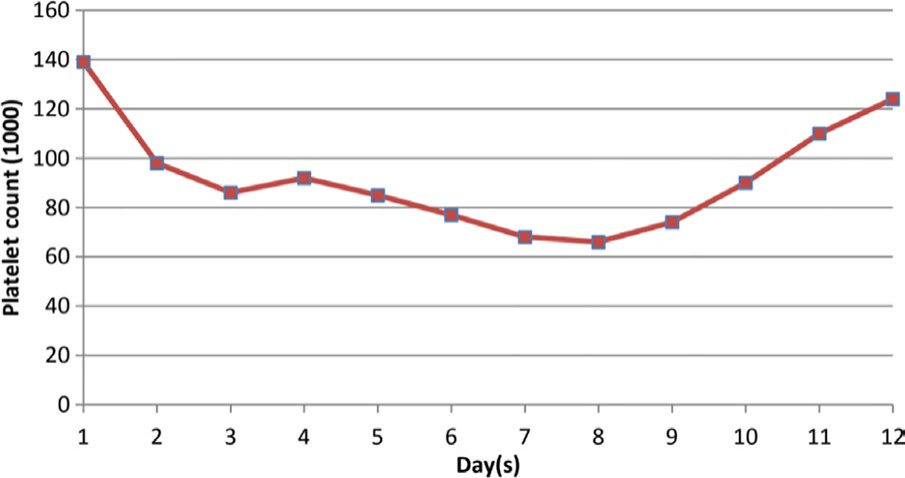
Trend of platelet count over the course of hospitalization

**Graph 3:**
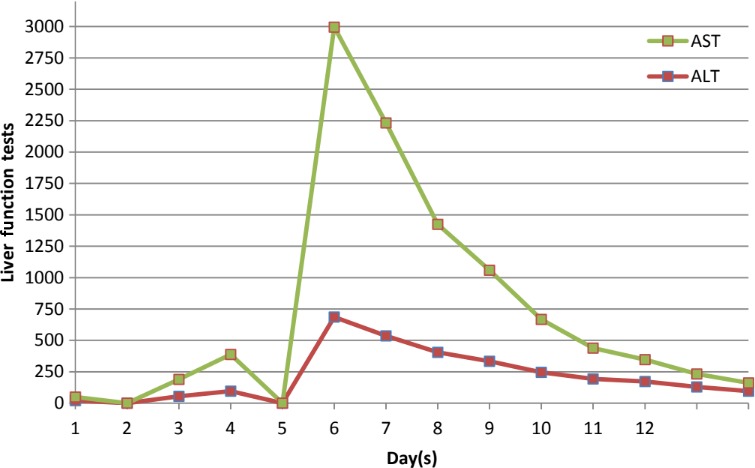
Trend of liver enzymes (AST and ALT) over the course of hospitalization

Acyclovir was started at the 10 mg/kg for every 8 hours. After initiation of acyclovir, patient's liver function tests and symptoms improved. Graphs 1, 2, 3 show improvement of AST, ALT, WBC, and PLT after initiation of acyclovir on day 5.

Her fever subsided thereafter and the thrombocytopenia and leukopenia resolved. The patient was discharged home in stable condition with total of 3 weeks of acyclovir infusion treatment and outpatient follow up. To date, the patient is doing well and is free of symptoms.

## 2 DISCUSSION

Herpes simplex virus commonly causes mucocutaneous infections in the general population. Hepatitis, as a complication of HSV, is rare phenomenon that represents <2% of viral hepatitides and is most prevalent in the immunocompromised or pregnant women following oro‐genital infection.^1^ This can rapidly progress to fulminant disease with declining liver function and cytopenias and can be fatal if left undiagnosed or untreated.^2^ Management consists primarily of intravenous acyclovir and supportive care. We present a case of fulminant herpes hepatitis in a woman following surgical instrumentation of the abdomen and successful treatment.

Since herpes hepatitis is rare and can present with nonspecific signs and symptoms, it can lead to being a missed diagnosis.^3^, ^4^ Patients with herpes hepatitis may not exhibit warts or candidiasis.^5^ Typically, herpes hepatitis is identified by fever, leucopenia, and elevated transaminases (about 100‐ to 1000‐fold above normal with AST predominance.^6^ Most cases show elevated transaminases and low to normal bilirubin, thus getting the name of “anicteric hepatitis”.^5^ Most patients who develop herpes hepatitis are immunocompromised (pregnant, newborns, on oral steroids, burn victims, organ transplantation, malignancy, or AIDS). However, about 25% of patients in a retrospective analysis of herpes hepatitis cases were immunocompetent prior to onset of herpes hepatitis.^6^

It is thought that the mechanism for developing herpes hepatitis would be any of the following: (a) A large HSV amount is transmitted on initial infection that overwhelms the host's immune system; (b) Dissemination from mucosal herpetic lesions because of defective macrophages, cytotoxic T lymphocytes, and delayed‐type hypersensitivity reactions; (c) Reactivation of a latent HSV or reinfection with a new strain of HSV 4 (Hepatovirulent HSV).

A liver biopsy is the gold standard for diagnosing herpes hepatitis though it is very invasive and can lead to complications in the setting of the coagulopathy.^7^ Transjugular liver biopsy would have the least bleeding risk.^6^ A liver biopsy would show hepatic necrosis, HSV, immunoreactivity to HSV, and intranuclear inclusions.^4^, ^7^ If a liver biopsy is not safe for the patient due to coagulopathy, then HSV PCR in real time would be another test to help diagnose herpes hepatitis.^3^, ^8^, ^9^ The study done by Beersma et al^10^ showed improved early diagnosis and management of herpes hepatitis. The study further showed liver kinetics and HSV DNA load in serum can lead to diagnosis. In contrast, our case was diagnosed with HSV DNA present in ascitic fluid.

Both HSV types (HSV‐1 and HSV‐2) can cause fulminant acute hepatitis.^9^ Herpes hepatitis represents <1% of all acute liver failure cases and <2% of all viral causes of acute liver failure.^3^ Nevertheless, it is very important to treat herpes hepatitis early and aggressively with acyclovir because it has an 80%‐90% mortality rate if left untreated.^5^ Acyclovir is a relatively safe treatment,^4^ and should be started as an empiric treatment when clinical suspicion is high. Vidarabine has also been used as a treatment for herpes hepatitis.^7^

Given that most cases of herpetic hepatitis occurs in the immunocompromised or pregnant population, this is an unusual case of the disease in an otherwise healthy woman. Nonspecific gastrointestinal symptoms and ascites coupled with objective data for sepsis has a relatively large differential. A viral etiology for this clinical scenario may not always be readily apparent. In the setting of recent abdominal instrumentation in sexually active individuals, our case illustrates the importance of clinician awareness of the herpes virus as a potential pathogen. Physicians must maintain a high index of suspicion for herpes hepatitis especially in patients who develop fever, ascites, cytopenia, and transaminases with AST predominance postoperatively. HSV DNA load with liver kinetics can lead to early diagnosis. Early initiation of acyclovir in the appropriate patient population is lifesaving given the high mortality of disease.

## CONFLICT OF INTEREST

None of the authors have any personal bias to declare.

Informed consent: Informed consent was obtained from the patient for educational use of the below mentioned data and no personal patient information has been disclosed.

## AUTHOR'S CONTRIBUTION

M. Yunce, P. Bhat, and D. Jaganathan completed the background research, drafted and edited the manuscript. M. Bahrain edited and completed the manuscript. P. Bhat completed all necessary revisions.
